# Immune Correlates of COVID-19 Control

**DOI:** 10.3389/fimmu.2020.569611

**Published:** 2020-09-29

**Authors:** Bhawna Poonia, Shyam Kottilil

**Affiliations:** Institute of Human Virology, University of Maryland School of Medicine, Baltimore, MD, United States

**Keywords:** COVID-19 control, immune correlates of COVID-19, COVID-19 vaccine, COVID-19 immune therapy, SARS-CoV2 specific immunity, COVID-19 B cells, SARS-CoV2 antibodies, SARS-CoV2 T cells

## Abstract

COVID-19 caused by SARS CoV2 emerged in China at the end of 2019 and soon become a pandemic. Since the virus is novel, pre-existing CoV2-specific immunity is not expected to exist in humans, although studies have shown presence of CoV2 cross-reactive T cells in unexposed individuals. Lack of effective immunity in most individuals along with high infectiousness of the virus has resulted in massive global public health emergency. Intense efforts are on to study viral pathogenesis and immune response to help guide prophylactic and therapeutic interventions as well as epidemiological assessments like transmission modeling. To develop an effective vaccine or biologic therapeutic, it is critical to understand the immune correlates of COVID-19 control. At the same time, whether immunity in recovered individuals is effective for preventing re-infection will be important for informing interventions like social distancing. Key questions that are being investigated regarding immune response in COVID-19 which will help these efforts include, investigations of immune response that distinguishes patients with severe versus mild infection or those that recover relative to those that succumb, durability of immunity in recovered patients and relevance of developed immunity in a cured patient for protection against re-infection as well as value of convalescent plasma from recovered patients as a potential therapeutic modality. This is a broad and rapidly evolving area and multiple reports on status of innate and adaptive immunity against SARS-CoV2 are emerging on a daily basis. While many questions remain unanswered for now, the purpose of this focused review is to summarize the current understanding regarding immune correlates of COVID-19 severity and resolution in order to assist researchers in the field to pursue new directions in prevention and control.

## Introduction

**FIGURE 1:**
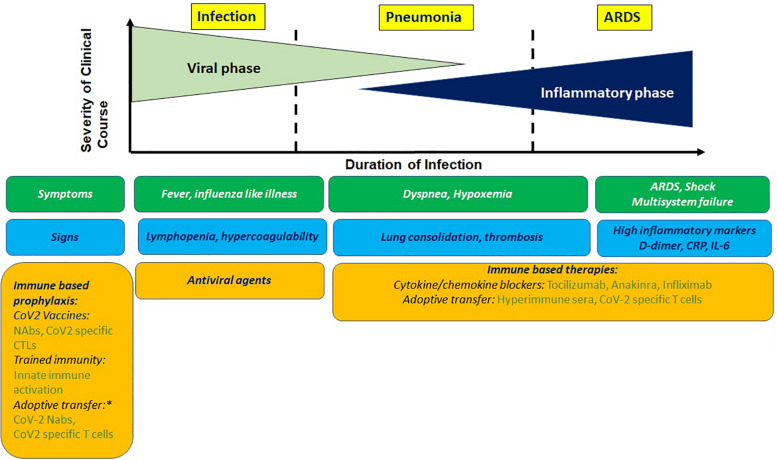
Clinical presentation of COVIO-19 with antiviral and immune based intervention stages. Prophylactic immune based approaches being tested involve stimulating innate or adaptive immunity or adoptive transfer of imrrune effectors. Potential therapeutic approaches involve blocking cyokine mediators of hyperinflammation and adoptive transfer of immune effectors. NAbs, neutralizing antibodies; CTL, cytotoxic T cells. *For high risk group.

Clinical presentation of COVID-19 ranges in signs and symptoms from asymptomatic to acute respiratory distress syndrome (ARDS; Figure 1). The mean incubation time is estimated to be ∼5 days after exposure. After an initial prodromal phase that lasts up to 10 days where maximal virus shedding occurs 5–8 h prior to symptom initiation, most infected individuals recover. A proportion of infected individuals progress to develop pneumonia, the average onset time for which is 9 days, and present with debilitating respiratory symptoms. Viral shedding in these individuals is prolonged and induces a host inflammatory response associated with increased levels of proinflammatory cytokines, which overwhelms antiviral immunity and lead to progressive, often fatal inflammatory sepsis and ARDS. These findings were confirmed very early during the pandemic from clinical data available from Wuhan (1).

The host immune system is known to play role in both protection from and pathogenesis of respiratory corona virus infections (2). Early evidence suggests that the host immune system is a double-edged sword in COVID-19. Early induction of protective immune responses are likely to ameliorate clinical severity of the disease while inflammatory cytokine response is likely to contribute to disease pathogenesis and mortality. The common features identified in multiple studies so far confirm the following immune changes in case of severe infection: lymphopenia, higher neutrophil-lymphocyte ratios, reduced percentages of CD8^+^, NK, CD4^+^, and B cells, exhausted lymphocytes with compromised functional response and cytokine storm. At the same time resolution of COVID-19 is associated with activated cellular and humoral responses, particularly presence of antigen specific CD4^+^ T cells in vast majority of COVID-19 patients that correlate with levels of antigen specific antibody response. We will review the most important studies that identified these features (summarized in Table 1) and discuss how they advance our understanding of role of immunity in COVID-19 control. We also summarize promising immune based prophylactic and therapeutic approaches that can potentially lead to COVID-19 control.

**TABLE 1:** Immune correlates of COVID-19 severity.

**Immune marker**	**COVID-19 case**				
	**vs healthy**	**Mild**	**Moderate**	**Severe**	**References**
Lymphocyte counts	Low counts	low	low	Very low	(3, 4)
Neutrophil counts	High counts	high	high	High, Neutrophil extracellular traps (NET)	(4)
NK cells	Low counts	Low counts	Low counts	Very Low counts, Increased NKG2A, Low CD107a, IFNγ, IL-2, Granzyme B and TNFα	(7, 8)
CD4 + T cells	Low counts, CoV2 S specific cells in all patients versus in 60% healthy controls	Functional cells present	Functional cells present	Global CD4 + cells hyperactivated, low IFNγTNfα producing polyfunctional	(3, 5–8, 11–14, 27, 28, 30)
CD8 + T cells	Low counts, CoV2 specific cells in most patients, no data on healthy	No exhaustion	No exhaustion	Global CD8+ T cells exhausted, NKG2A+, CD107a, GrB producing high, low PD-1^–^TGIT^–^CTLA4^–^	(3, 5, 6, 8, 13, 14, 28)
Follicular helper T cells	Activated	Activated	Activated	ND	(5)
B cells	Activated	Increased plasma cells	Increased plasma cells	Decreased plasma cells.	(5, 8, 9, 18, 46)
IgG/IgM/IgA	Present	Present	Present	Present	(5, 34–45, 47)
Cytokines	High in severe COVID	Low	Low	Cytokine storm	(3–5, 12, 15, 16, 18–26)

*ND, no data.*

### Antigen Non-specific Immune Response in COVID-19

Initial studies focused on global immune cells in COVID-19 and identified immune features that correspond with pathogenesis. Severe cases typically have lower lymphocytes counts, higher leukocytes counts and neutrophil-lymphocyte-ratio (NLR), as well as lower percentages of monocytes, eosinophils, and basophils (3, 4).

Among the first reports on immune response generated in a COVID-19 patient was a case report in an otherwise healthy 47 years old female who presented with mild-to-moderate symptoms of COVID-19 and achieved clinical resolution without antiviral treatment (5). In this case, normal counts of blood lymphocytes and neutrophil levels along with elevated blood C-reactive protein were observed. Importantly, a simultaneous peak increase in IgM, IgG, plasma B cells, activated follicular helper T cells and activated CD8^+^ and CD4^+^ T cells expressing HLA-DR and CD38 in the blood on days 7–10 of onset of symptoms occurred. This study was the first of its kind to suggest that SARS-CoV2 infection induced a broad adaptive immune response that may have facilitated in viral clearance and clinical recovery. Whether these immune changes are present in most resolved cases and how they differ in those on spectrum of clinical symptoms ranging from asymptomatic to extremely ill, will help in further understanding the importance of these immune correlates of COVID-19 control in infected subjects.

In a larger study of 65 patients in Wuhan that were classified based on mild, severe and extremely severe illness, multiple immune changes were differentially present in mild versus severe cases (6). The absolute numbers of CD4^+^ T cells, CD8^+^ T cells and B cells decreased with increasing severity while their activation status went higher. Presence of hyperactivated T cells associated with severe illness; expression of activation markers HLA-DR and CD45RO on CD4^+^ and CD8^+^ T and the percentages of IFNγ producing CD8^+^ and CD4^+^ T cells were increased in extremely severe cases. Because IFNγ producing T cells are expected to be antiviral, it is not clear why higher percentages would associate with poor outcomes. Indeed, another study of 68 patients in China found that the cytotoxic CD8^+^ T cells and NK cells were exhausted in function during SARS CoV-2 infection (7). In patients with severe disease lower CD8+ T cell and NK cell counts were observed compared with counts in mild cases. Here, exhausted status of these lymphocytes was reflected in increased expression of inhibitory receptor NKG2A that results in functional exhaustion on these cytotoxic cells. Further, NK cells and CTLs from severe cases had reduced capacity to produce cytotoxic effector molecules CD107a, IFNγ, IL-2, Granzyme B, and TNFα. Importantly, in patients convalescing after therapy, numbers of these lymphocytes go up along with a reduction in NKG2A expression. Authors concluded that antiviral immunity during COVID-19 is compromised during early stage and downregulation of inhibitory NKG2A may associate with disease control. These two studies don’t correspond with respect to status of cytokine producing lymphocytes in severe cases and highlight the need for more data from other labs to get a coherent picture of role of IFNγ producing cytotoxic cells in the disease pathogenesis.

The role of B cells in COVID-19 pathogenesis is not clear. While increased plasma cells are reported in mild cases (5, 8), in case reports of patients with no B cells (agammaglobulemia) COVID-19 was mild and these patients recovered without treatment (9).

Functional impairment of effector T cells was associated with severe outcome of COVID-19 in another study. In 16 COVID-19 patients, comparison of mild and severe cases of disease identified CD4^+^ T cell functional defects and CD8^+^ T cell exhaustion associated with severe outcome (10); lower expression of TNFα and IFNγ producing CD4^+^ T cells and higher levels of killer CD8^+^ T cells expressing Granzyme B and perforin were present in severe cases. Lower frequencies of IFNγ producing CD4+ T cells in severe COVID-19 were confirmed in another study (3, 11). Detailed characterization of CD4+ T cells from patients with COVID-19 pneumonia showed T cell activation, senescence and exhaustion along with a skewing toward Th17 type (12). These studies indicate a role for polyfunctional CD4^+^ T cells in COVID-19 control. Recent studies have in fact identified presence of virus specific CD4^+^ T cells in most COVID-19 patients and the levels of these cells correlate with virus specific IgG (13, 14), indicating helper CD4^+^ T cell response may have an important role in limiting this infection. The importance of CD8^+^ T cells in pathogenesis or protection is not clear. High expression of perforin and granzyme B in severe COVID-19 cases could suggest some role for host immune response in exacerbating disease. Patients with severe COVID-19 had lower proportion of CD8^+^ T cells with “non-exhausted” phenotype PD-1-TGIT- CTLA4-, which may indicate CD8^+^ T cells getting activated and exhausted in more severe cases, limiting their effector potential.

A comprehensive analysis of immune cell phenotypes and gene expression signatures identified features of early recovery stage (ERS) or late recovery stage (LRS) of COVID-19 (8). Early recovery stage patients had increased plasma cells and decreased naïve B cells like in previous study. In ERS patients increased monocytes as well as CD4^+^ and CD8^+^ T cells with inflammatory gene signature was present compared with patients with LRS. This signature may represent residual inflammatory response with COVID-19. Several novel B cell-receptor (BCR) changes, such as IGHV3-23 and IGHV3-7, and isotypes (IGHV3-15, IGHV3-30, and IGKV3-11) previously used for virus vaccine development were confirmed. The strongest pairing frequencies, IGHV3-23-IGHJ4, indicated a monoclonal state associated with SARS-CoV-2 specificity, providing further clues for vaccine development.

### Proinflammatory Cytokines and Chemokines in COVID-19 Pathogenesis

While inflammation is necessary for antiviral immune response, a clear sign of an immune system in overdrive being detrimental for COVID-19 is the observation of “cytokine storm” in blood of critically ill COVID-19 patients manifested as persistent high fevers, severe respiratory distress, and lung damage (15). Some severe COVID-19 cases present with elevated proinflammatory cytokines (3, 15, 16), while in the case of a recovered COVID-19 patient, minimum proinflammatory cytokines were induced indicating milder course of infection is associated with less systemic effects (5).

A signature of proinflammatory cytokines associated with severe disease outcome with COVID-19 is emerging. Serial detection of IP-10, MCP-3, and IL-1RA in 14 severe cases showed that the continuous high levels of these cytokines were associated with increased viral load, loss of lung function, lung injury, and fatal outcome (17). Other studies have found elevated levels of IL-6 and IL-1β, as well as IL-2, IL-2R, IL-8, IL-10, IL-17, G-CSF, GM-CSF, CXCL10, MCP1 and MIP1-α, and TNF associate with severe outcome, while elevated ferritin and IL-6 were also linked to mortality (4, 18–20).

COVID-19 pneumonia patients had elevated levels of multiple plasma cytokines and chemokines relative to healthy controls; these include Galectin 1, 3, 9, 10, CCL2, 3, 4, CXCL6, MICA, GITR, TNF, IFNγ, PD-L1, IL-1α, IL-1β, IL-4, IL-6, IL-7, IL-8, IL-10, and IL-13 (12). In patients with severe respiratory failure and ARDS, immune dysregulation characterized by sustained TNF and IL-6 production and IL-6-mediated low HLA-DR expression as well as lymphopenia was reported (21).

SARS-CoV-2 is a respiratory pathogen and immune response generated in the lungs is more likely to inform the role of immune mediators in pathogenesis. Local immune response in bronchoalveolar lavage (BAL) of COVID-19 patients presents a picture of inflammatory mediators that distinguishes this infection with previous SARS or non-viral pneumonia. Uniquely, SARS-CoV-2 induced IFN stimulated genes exhibiting immunopathogenic potential, with overrepresentation of genes involved in inflammation (22). Further, various neutrophil chemotractants were induced, explaining observations of high levels of neutrophils in COVID-19. In severe COVID-19, the levels of neutrophils are further higher as well as these neutrophils were shown to release chromatin, microbial proteins and oxidative enzymes resulting in formation of structures called neutrophil extracellular traps (NETs), which may contribute to respiratory failure (23). A transcriptomic signature of elevated proinflammatory genes including CXCL10, MCP1, MIP1A, MIP1B in BAL of COVID-19 patients has been confirmed by others (24). A multi-omics systems biology study performed a comprehensive evaluation of immunity to COVID-19 infection in two different cohorts in Atlanta and Hong Kong (18). Common alterations were reduced expression of HLA-DR and proinflammatory cytokines in myeloid cells from patients, increased plasma levels of inflammatory mediators, impaired mTOR-signaling and IFNα production by pDCs. Single cell transcriptomics revealed clusters of T cells and monocytes characterized by expression of ISGs. Role of bacterial products possibly of lung origin in augmenting the inflammatory cytokine response in severe COVID-19 was indicated here as enhanced levels of bacterial DNA and LPS in plasma positively correlated with plasma levels of EN-RAGE, TNFSF14, OSM, and IL-6.

An obvious relevance of these studies is identification of potential targets to pursue for mitigating these detrimental effects of excessive inflammation. Importantly, treatment with IL-6 receptor blocking antibody tocilizumab in a clinical trial ChiCTR2000029765 has showed clinical benefit in 21 severely ill patients (25), suggesting neutralizing mAbs against other pro-inflammatory cytokines may also be of use. Other potential targets in this regard include TNF, IL-1, IL-17, and their respective receptors (26).

### SARS-CoV2 Specific T Cell Immunity in COVID-19 Control

With the availability of SARS-CoV2 specific reagents including HLA restricted peptide pools becoming available, more recent studies have been able to identify viral epitopes targeted by the immune response.

In an elegant study, Grifoni et al., identified presence of SARS-CoV-2 specific CD4^+^ and CD8^+^ T cells in circulation of most COVID-19 convalescent patients (14). With the help of HLA class II predicted peptide megapools they demonstrated that all COVID-19 patients had robust viral spike glycoprotein (S) specific functional CD4^+^ T cell response that correlated with magnitude of SARS-CoV2 specific IgG and IgA titers. Most investigational vaccines target viral S protein and these results are among first to indicate usefulness of the approach. An important finding from the study was that upto 60% individuals unexposed to SARS-CoV-2 also harbored S specific CD4^+^ T cells and a smaller proportion also had virus specific CD8^+^ T cells. The authors discussed that this potentially indicates presence of cross-reactive immune response between this virus and seasonal coronaviruses. In subsequent study, the same group mapped 142 T cell epitopes across CoV2 genome to interrogate CD4+ T cell repertoire and showed presence of pre-existing memory CD4+ T cells that are cross-reactive with comparable affinity to CoV2 and common cold coronaviruses HCoV-OC43, HCoV-229E, HCoV-NL63, or HCoV-HKU1. the It remains to be seen whether these responses have relevance for protection against COVID-19 exposure in these individuals (27).

Another important study corroborated these findings and demonstrated presence of SARS-CoV2 S protein reactive CD4^+^ T cells in 83% COVID-19 patients and in upto 34% seronegative healthy controls (13). In this preprint, authors used overlapping peptide pools corresponding to either C or N terminus of S glycoprotein and measured antigen specific cells by enumerating frequencies of CD4^+^ T cells that co-expressed CD40L and 4-1BB upon stimulation with either peptide pool. Using this approach an important difference in the region of spike protein targeted in patients versus controls was discovered. Patient CD4^+^ T cells targeted both N and C terminal of S protein almost equally, whereas CD4^+^ T cells from healthy controls predominantly targeted C terminus of S glycoprotein, which incidentally has high homology with S protein of “common cold” coronaviruses and does not contain the receptor binding domain. Another distinguishing feature between virus specific CD4^+^ T cells from patients was evidence of their recent *in vivo* activation, indicated by expression of CD38, HLA-DR and Ki67 markers and an effector memory signature indicated by co-expression of CD38 and HLA-DR. These diverse characteristics of S specific T cells in patients and healthy individuals raise important questions regarding their potential protective versus pathogenic function in a healthy individual when exposed to SARS-CoV2. Further, levels of these S reactive CD4^+^ T cells can modulate outcome of infection with CoV2.

Others have also shown presence of COV-2 reactive T cells recognizing N protein in unexposed individuals (28). In 19 out of 37 unexposed individuals tested, presence of IFNγ producing T cells in response to CoV-2 N, NSP7 and NSP13 peptide stimulation was shown using ELISPOT assays. Further characterization of CD4 T cells in unexposed individuals showed reactivity to an epitope of N protein that as high homology with N protein of MERS-CoV, OC43 and KHU1 and this is also present in individuals that recovered from SARS and COVID-19.

All these data suggest there are potentially cross-reactive T cells to various betacoronaviruses of humans and possibly animals that are present in individuals unexposed to CoV2.

Airway memory CD4^+^ T cell mediate protective immunity in respiratory coronaviruses (29) and it will be of particular interest to see if CoV2 specific CD4^+^ T cell in BAL can predict resolution of infection. A very unique aspect of COVID-19 pathogenesis is significantly low pathogenesis in younger people. Whether presence of cross-reactive CD4^+^ T cells can explain some of this divergent outcome remains to be seen. Longitudinal studies are required to for example examine their presence in young adults or children who get more exposed to common cold.

To what extent T cell response associate with serostatus and clinical course of COVID-19 was addressed by (30). Testing unexposed individuals, exposed family members and individuals with acute or convalescent COVID-19 this study showed highly activated CoV2 specific T cells with cytotoxic phenotype during acute phase, while convalescent stage T cells were polyfunctional with stem like memory phenotype. CoV2 specific T cells were present in seronegative exposed family members and convalescent individuals. These CoV2 specific T cells were present even in seronegative individuals, suggesting a possible non-redundant role of cellular and humoral responses in COVID-19 control.

### Role of B Cells and Antibodies COVID-19 Control

Most effective human vaccines work by generating neutralizing antibodies and SARS-CoV-2 S protein is target of most investigational vaccines. Animal studies have shown encouraging prophylactic results with several vaccine candidates. DNA vaccine candidates expressing SARS-CoV-2 S protein evaluated in rhesus macaque challenge model showed vaccine generated neutralizing antibody titers correlated with it’s protective efficacy (31).

Intranasal administration of replication-incompetent recombinant serotype 5 adenovirus, Ad5-S-nb2, carrying a codon-optimized gene encoding Spike protein (S) elicited systemic and pulmonary S specific antibodies and protected macaques challenged 30 days after vaccination (32). A single immunization with an Ad26 vector encoding S variants protected macaques challenged with SARS-CoV-2 by the intranasal and intratracheal routes; here also titers of neutralizing antibodies correlated with protective efficacy indicating antibody levels as an immune correlate of protection (33). Multiple human clinical trials are currently ongoing with various vaccine candidates and show neutralizing antibody generation in preliminary unpublished results (Table 2).

**TABLE 2:** Major phase 2/3 clinical studies testing COVID-19 vaccine candidates in humans.

**Modality-Name**	**Company**	**Study/Phase**	**Vaccine induced immune response**
mAb-REGN-CoV2 Antiviral antibodies	NIAID/Regeneron	III	Passive transfer of antibodies
mAbs-LY-CoV555 Antiviral antibodies	Eli Lilly/NIAID	III	Passive transfer of antibodies
mRNA-1273	Moderna	The COVE study/phase III	Anti-spike antibodies
Inactivated SARS-CoV2-AZD1222	AstraZeneca/Univ Oxford	II/III	Anti-spike antibodies, T cell response
mRNA-BNT162	Pfizer/BioNTech	II/III	Neutralizing antibodies
Inactivated vaccine	Wuhan Institute of Biological Products/Sinopharm	III	Neutralizing antibodies
Inactivated vaccine-CoronaVac	Sinovac	III	Neutralizing antibodies
Recombinant S protein-NVX-CoV2373	Novavax	IIb	Neutralizing antibodies; polyfunctional CD4 T cell

The importance of CoV2 specific antibodies in infected patients for pathogenesis or protection is to be determined. Most patients develop virus specific IgG and IgM within days to weeks of symptom onset, the relevance of which for clinical outcomes is being investigated (5, 34, 35). Antibody responses tend to be highest in those with severe symptoms, whereas those with mild cases have lower levels of neutralizing antibodies (36). This is a pattern seen with common cold coronaviruses where typically milder symptoms and lower Ab titers are present (37). A striking increase in frequencies of plasmablasts in peripheral blood of COVID-19 patients has been shown by several large studies (18, 38–40), although correlations with high levels of RBD specific IgM and IgG were not confirmed. Sequencing of antibody repertoire showed severe COVID-19 patients have oligoclonal expansion of B cells with antibodies enriched for long and divergent CDR3 sequence (38).

Whether antibodies protect from re-infection is one of the most critical questions that will decide management of this pandemic. Animal studies with rhesus macaques showed neutralizing antibodies developed during primary SARS-CoV2 infection and protected the animals from a re-challenge with an identical viral strain (41, 42). Whether patients that recover from COVID-19 develop protective antibody response that prevents re-infection is still to be determined. It will also depend on the durability of antibody response and the variation of the new infecting virus. Original case reports of recovered people getting re-infected with SARS-Co-V2 raised concerns regarding development of memory immune response (43), though these cases turned out to not carry infectious virus and thus reflected a positive PCR test due to amplification of inactive or dead viral genetic material and not reactivation or re-infection. The first confirmed re-infection was reported on August 24, 2020 in a 33 year-old man from Hong Kong who first got infected with SARS CoV2 in March and then four and a half months later got re-infected while traveling to Europe. This raises questions about the durability of immune protection in recovered individuals. Several studies have shown that neutralizing antibodies begin to wane at about 3 months after infection (37, 44). The IgM against RBD of S protein and N protein became undetectable at about 12 weeks in most patients who recovered, however IgG-S/N had a contraction phase which was followed by relatively high stabilized levels in most individuals at 6 months follow up (45). Further long-term studies are needed to describe antibody decay rates beyond the 90 days as well as to understand the mechanism for lower persistence of antibodies in convalescent samples. While many non-persisting viruses such as measles, mumps, rubella and vaccinia induce long-lasting antibody response, other like influenza induce antibodies with short half-life. Many seasonal coronaviruses and SARS-CoV1 induce short lived antibody response (46) and CoV2 appears to induce a similar short-lived response.

Also relevant for understanding antibody mediated protection is to know the titers of antibody that are in fact required for sustained immunity, which is not yet known in case of COVID-19. It is entirely possible that the low levels present after 12 weeks are still protective and will be known in due time.

Besides persisting low levels of antibodies another player to consider when thinking about long-term protection is memory lymphocytes. During resolution of a viral infection even when antibody levels wane in absence of antigen, there are memory B cells that linger in bone marrow until a re-infection occurs, at which time they can differentiate into plasma cells and produce antibodies. The data on status of memory B cells during COVID are still lacking. However, persisting memory B cells in individuals with mild symptomatic recovered COVID-19 infection (47) suggest possible long-term sustenance of virus specific B cell response.

There is also evidence that some neutralizing antibodies identified from 2003 SARS outbreak can neutralize SARS-CoV2 in patients. Thus continued discovery of more potent neutralizing antibodies in recovered COVID-19 patients will provide basis for developing antibody therapeutic modalities (48).

### Lessons From Immune Response in Previous Coronavirus Infections

SARS-CoV2 belongs to betacoronavirus genus that contains five pathogenic human coronaviruses. Besides the human coronaviruses that cause the “common cold” viz., OC43 and HKU1, the coronaviruses that have arisen through zoonosis and cause severe diseases in humans are SARS-CoV, MERS-CoV, and SARS-CoV2, which emerged in 2003, 2012, and 2019, respectively. As with other coronaviruses, the spike glycoprotein (S) homotrimer on the CoV2 surface plays an essential role in receptor binding and virus entry. There is high level of similarity in the amino acid sequence of structural proteins of these CoVs whereas the accessary proteins are more unique (49). The most unique characteristic of this CoV2 is its very high transmissibility and infectiousness, the structural basis for which currently under investigation. Thus, the immune response generated against previous coronaviruses may not be the perfect template to work off for this virus. Investigations of SARS CoV1 and MERS pathogenesis do, however, provide indications about role of specific immune cells in resolving human coronavirus infections. These studies have indicated T cell immunity plays role in resolution of infection (50). Both MERS and SARS CoV1 recovered patients have long lasting memory T cell response. This contrasts with lack of anamnestic B cell response and the results have implications for protection from re-infection. In patients that recovered, SARS-CoV1-specific antibody response is short lived. In these patients, virus-specific IgM and IgA response lasted less than 6 months, while virus-specific IgG titer peaked four-months post infection and markedly declined after one year. The antigen specific memory B cell responses were completely undetectable at 6 years after SARS infection (46).

Despite the lack of virus-specific memory B cell response, SARS-CoV1-specific memory T cells persist in recovered patients for up to 6 years post-infection, whereas there was no such specific response in either close contacts or healthy controls. Important role for memory T-cells in long-term protection against SARS-CoV1 infection has been established by others (29, 50–52). Severe SARS-CoV1 infection in humans was characterized by the delayed development of the adaptive immune response and prolonged virus clearance (53). Decreased numbers of T cells strongly correlated with the severity of acute phase of SARS disease in humans (54). Pathological investigation of patients with lethal SARS reveals acute pulmonary edema, extensive inflammatory cell infiltration, multi-organ failure, thromboembolic complications, and septicemia (55). Severe lung and systemic inflammation is believed to result from cytokine dysregulation; in patients with SARS elevated levels of cytokines such as TNF, CXCL10, IL-6, and IL-8 likely contributed to the poor outcome (55), strikingly similar to cytokine storm reported in severe COVID-19 patients. A study of these immune features in CoV-2 infection will inform comparative studies of immune landscape in COVID-19. Much progress has been made regarding detailed characterization of CD8^+^ and CD4^+^ T cell epitopes involved in SARS CoV1 immunity. For example, polyfunctional CD8^+^ T cells (SSp-1, S978, and S1202 specific) were identified in surviving patients over one year post-infection (56). Memory CD4^+^ T cells specific for epitopes within the S protein of SARS-CoV were identified in recovered individuals (57). Reagents for such in depth characterization of T cell responses to CoV-2 are becoming available and long term follow up studies will identify durability of T cell response in COVID-19.

Recent studies have shown presence of long-lived cross-reactive T cells in that recognize multiple betacoronaviruses (27, 28). Presence of SARS N protein specific T cells were shown in recovered individuals 17 years after the 2003 SARS infection; importantly these T cell had strong cross-reactivity to SARS CoV2 N protein. How much such cross-reactive T cells impact susceptibility to COVID-19 or the extreme heterogeneity observed in severity remains to be seen and has potential implications for management of the current pandemic.

### Immune Dysregulation as Predisposing Condition for COVID-19 Progression

COVID-19 is more severe in those with aged immune system (the elderly) and in those with co-morbidities that dysregulate or suppress immunity including cancer and potentially in chronic infections like HIV. This argues that defects in antiviral host defense mechanisms impact COVID-19 pathogenesis, although definite studies on this are still in future. On the other hand, it remains to be seen whether a blunted immune system will result in less likelihood of hyper inflammatory condition like cytokine storm. A multicenter study of 105 cancer patients and 536 age-matched controls provided evidence for cancer being a significant underlying condition that results in more severe outcome with COVID-19 although immune response in these patients was not studied (58). Here, those with hematologic, lung or metastatic cancers had most severe outcomes while those with non-metastatic cancer had similar COVID-19 pathogenesis as non-cancer patients. Few preliminary reports have presented COVID-19 in HIV infected patients (59), and clinical features of COVID-19 in other immunosuppressed populations require more appropriate larger studies.

## Current Vaccines and Therapeutics

Currently there is no proven therapeutic or vaccine to control COVID-19 and many approaches are being tested in multiple clinical trials. The Sputnik V vaccine developed by Gamaleya research Institute Moscow is the first COVID-19 prophylactic that has been approved for use in humans. This vaccine candidate however did not enter phase III clinical trials to assess efficacy and as such concerns about it’s safety and efficacy are raised.

While a clearer understanding of immune correlates of clearance or protection from SARS-CoV2 will develop in near future, investigations so far have provided a definite correlation between certain immune responses and control of this infection, which evoke confidence that stimulating these responses will provide protection. At the same time identifying immune correlates of severe pathogenesis such as proinflammatory cytokines facilitate development of effective molecules to reverse these detrimental immune responses.

In the first indication that vaccine can train immune system against SARS-CoV2, neutralizing antibodies developed in all eight people who took the experimental Moderna vaccine mRNA-1273. Similarly, Novovax S protein mRNA-based vaccine elicited robust neutralizing antibody response similar or superior to that seen in human convalescent sera and phase III trials for this have begun.

Another prophylactic approach being tested is use of neutralizing antibodies that bind host cellular receptor ACE2 as shield against the virus (60). Multiple corporations are testing various combinations of such antibodies identified from patients that recovered from COVID-19 for future clinical trials. These include COVI-SHIELD from Sorrento containing mixture of three antibodies that combined recognize three specific regions of the SARS-CoV-2 Spike protein; GSK and Vir candidates VIR-7831 and VIR-7832, and antibodies from Regeneron, Eli Lilly, Celltrion. If successful these can provide prophylaxis for high-risk populations and a post-exposure therapeutics. Currently multiple trials are on to assess the efficacy of these and other vaccine modalities for preventing COVID-19. Table 2 shows leading candidates in late-stage clinical trials.

Finally, it is suggested that boosting of non-specific innate immune responses also termed as “trained immunity” may be explored for inducing heterologous protection against COVID-19. Certain live vaccines like Polio, BCG and measles induce epigenetic, transcriptional and functional reprogramming of immune cells (61–63) that generate a cross-reactive non-specific innate immune response against unrelated pathogens. On the therapeutic side, association of cytokine storm with severe disease has led to testing of blocking compounds against these cytokines. Among the promising biologic therapeutics are Tocilizumab (anti-IL-6), Anakinra (anti-IL-1), and Infliximab (anti-TNF) (25, 26).

## Conclusion

Finding immunological mediators of CoV2 pathogenesis provides obvious cues to developing interventions against the virus. While most infected individuals develop neutralizing antibodies against CoV2 spike protein, there is clear association between antibody titers and severity of the disease. Whether this is associative or causative remains to be determined. Then, there is the question of antibody durability. In COVID-19 convalescent individuals antibody levels wane within three weeks. This raises questions about effectiveness of antibodies in protection from re-infection and for management strategies like COVID-19 recovered status as measure of “immunity passport.” Most efforts regarding vaccine development against COVID-19 are focused on generating neutralizing antibodies against viral spike protein. Animal vaccine challenge experiments are encouraging and have shown clear protective effect of these neutralizing antibodies. Many human clinical trials show leading vaccine candidates induce strong neutralizing antibody response. Whether vaccine induced antibody response is durable is not yet known. The correlations between defective virus specific T cell response and disease severity indicates cellular immunity has a potential protective role in addition to antibodies. Ongoing studies will refine these correlates of control as well as answer important questions such as immunological reasons behind asymptomatic infections, low pathogenesis of this virus observed in young adults and children compared with elderly and role of pre-existing cross reactive or trained immunity in the observed varied pathogenesis across regions and populations of the world.

## Author Contributions

All authors listed have made a substantial, direct and intellectual contribution to the work, and approved it for publication.

## Conflict of Interest

The authors declare that the research was conducted in the absence of any commercial or financial relationships that could be construed as a potential conflict of interest.
